# In Nature's School

**Published:** 1889-07-27

**Authors:** 


### The Doctor's Pulpit.

### IN NATURE'S SCHOOL.

### Dewdrops.

"Ilka blade o' grass keps its ain drap o' dew." Every student of the most elementary science is familiar in these days with what, for want of a better word, may be called the "machinery" of dew. They know that the rain which has fallen, and been drunk in by the thirsty ground, is given up again more or less quickly as vapour. During the day, when the air is warmed by the sun, the vapour passes away into the higher regions of the atmosphere, and after that is saturated, the remainder forms clouds. But as the sun goes down and the air near the surface of the earth becomes sufficiently cooled to condense the vapour as it rises, there is an arrest of its upward course, and a formation of dew, which is deposited in the most beautiful crystal drops on leaf and flower and myriad blades of grass. The Scotch ballad-poet who wrote the musical line which we have quoted at the commencement was struck by the wonderfully uniform distribution of the refreshing drops as they were formed in the silent laboratory of Nature. He had noticed, perhaps thousands of times, that whether there was much dew or little on any particular evening, it was practically impossible to find even a single blade of grass which remained perfectly dry. The same phenomenon must have struck other people who are in the habit of taking evening walks with their eyes open. So universally understood is this that no one thinks the poet has indulged his professional license in the least when he has said that every single grass-blade obtains its own crystal drop when the dew falls.

Carlyle speaks of Nature as a "beneficent mother;" and this miracle of the formation and equal distribution of the dew is one of a series of phenomena which all go to shew the steady, orderly, and constant provision which is made for all kinds of creatures that breathe and live on the earth's surface. In one short country walk in the evening it is astonishing what a variety of vegetable and animal life may be observed. Twenty different kinds of birds can be seen flying, or heard uttering their peculiar cry in bush or tree; butterflies of varying species and colour; moths, and others of their kind, innumerable; beetles "wheeling their droning flight"; mice and moles in the field or hedgerow; weazles and stoats with noiseless haste running down their prey, or escaping to the shelter of the bushes; hares with their ears erect and prompt flight to cover; rabbits bustling off at the first hint of possible danger; cattle browsing in the meadows; sheep gnarling on the hillside; horses resting after the work of the day; labourers trudging homeward with tired steps and sober looks; children playing in the village street or the shady lane; trees and shrubs, flowers and grass;—all these are the common objects of a summer evening walk. Every one of them, and all other animated things, draw their supplies from the breast of Mother Earth. The bare brown soil of the fallow field can convert itself or be converted into food, shelter, and pleasure for all these multitudinous forms of life. From the earthy elements that thinly cover the permanent rocks this infinity of living forms are evolved by what we call natural processes. They run their little course of months or years, and then are reconverted into earth; to issue forth after a time again into the same or other stages of animal or vegetable existence. This world-mill, which is always grinding, always showing new finished products, always, after a period more or less brief, reducing them to their elements again, is a standing miracle of order, uniformity, providence. In the larder of universal Nature there is food enough for all; in her vast clothing factory every creature may find the means of protecting itself from the summer's heat or the winter's cold.

Whence, then, is the misery of the world; whence the strife; whence the "battle, and murder, and sudden death"? There are certain insects which are particularly partial to the leaves of cruciferous plants, such as the cabbage or the turnip. In some seasons it seems as if all the insects of the country had swooped down upon one particular field. What is the result? The tender leaves of the young turnip disappear in a few days, or even hours, as if by magic, leaving the ground almost as bare as if it had never been sown. So much is this the case that the farmer in despair is often compelled to plough it up afresh and to sow his turnip seed over again. If the insects had had sense to distribute themselves over a hundred or a thousand fields instead of one, they would not have destroyed either the farmer's crop or their own means of subsistence. "Ilka blade of grass keps it ain drap o' dew"! Yes, but why? Because the grass spreads itself over every available foot of bare earth in the world. If all the grass-blades of Europe, Asia, Africa, and America had insisted upon living, say in Midlothian, the Scotch poet would not have been able to say with truth that every blade got its own crystal drop. There would not in that particular locality be nearly enough for the demands of all the universal grass family.

The application is surely quite plain. Up to the present time the world is amply sufficient for its population. The barns and storehouses of Nature, her vineyards and her orchards, are full. But men will insist upon gathering themselves into strictly limited spaces, like the insects which crowd together on a single cabbage-leaf, and consume it all in a day, leaving nothing but starvation for the morrow. Now whatever theories or sentiments may be held about love of country and emigration, nothing is more certain than that the number of fleas which can live on a single cabbage-leaf is strictly limited? As soon as the leaf is eaten, removal is imperative unless the fleas set to work to eat one another. Precisely thus is it with England, with Scotland, with London, with Manchester, with Glasgow. There will come a time when these industrial and populous cabbage-leaves cannot sustain a single additional insect. Even now the crowding is so great that men and women, if not actually devouring one another with their physical teeth, are struggling and fighting desperately with each other for breathing space, instead of standing shoulder to shoulder and co-operating in the general labour of increasing the fruits of the earth, and making an improved use of them. In such circumstances health, or anything approaching health, is impossible. Man was designed to be a co-operating animal ordinarily, and a fighting animal abnormally and occasionally. Nature did not make him a beast of prey. He has no carnivorous teeth, no serpent's poison fangs, no resistless weight to bear down opposition. When he fights he does not rush into the fray, like the lion, full armed and ready for the conflict. He has to make fighting tools and defences of all kinds, because Nature has left him bare and destitute of such. But why did she leave him bare and destitute of fighting teeth? Because she purposed something better for him than fighting. She purposed reasonableness, friendliness, brotherliness, co-operation.

When we crowd together in such vast hordes as we see in modern times, we frustrate the purpose of Nature. We render co-operation impossible, and force ourselves into strife. The absence of co-operation is the absence of health; the presence of strife is the beginning of all sorts of tragedies and miseries. The rich despise the poor, and the poor hate the rich. Men of the same class and station rush like twenty dogs all at once upon the same bone. The bone is not sufficient for more than one, and the nineteen who do not get it have nothing to do but fight with one another, or to devour the twentieth if they can catch him. Nature provides a drop of dew for every blade of grass; but in order that each several blade may have its full share there must be, as there is, a fairly uniform distribution of the grass over the whole of the available surface of the globe. Reason tells us that the Providence which provides for the wants of human creatures makes sufficient provision for all, but insists that those who lack food and other necessaries shall look for them where they are to be found. Health and common-sense alike demand that if the British cabbage-leaves are too crowded, and threaten to disappear, the British insects shall use their legs and wings and seek other cabbage-leaves elsewhere.

